# Supplementary material for: Interactive visualization of metric distortion in nonlinear data embeddings using the distortions package
Source: Brief Bioinform. 2026 Mar 30;27(2):bbag136. doi: 10.1093/bib/bbag136 (PMC13034850; doi:10.1093/bib/bbag136)
Supplement: disto-supplementary_bbag136 [file disto-supplementary_bbag136.pdf]

Supplementary Material

Interactive Visualization of Metric Distortion in Nonlinear Data Embeddings using the `distortions` Package

Kris Sankaran, Shuzhen Zhang, Chenab, Marina Meilă

1 Working with large-scale data

Single-cell studies now routinely profile millions of cells. Visualizing the associated nonlinear embeddings using scatterplots can use to severe overplotting, and the ellipse marks used by `distortions` potentially exacerbate this issue, since viewers are expected to compare the orientations and sizes of ellipses, rather than just cell-wise coordinates. For work with the `distortions` package, we recommend learning both the embeddings and the local metrics $\mathbf{H}^{(i)}$ using the full data, since both steps only require nearest neighbors graphs, which can be computed or approximated efficiently. For visualization, it is challenging to perceive differences in overplotted data, even with high-resolution screens.

Therefore, we recommend randomly sampling up to this many points in a final embedding plot. The associated ellipses still reflect estimates of the local metrics made using the full dataset. Alternatively, if interest lies in a particular trajectory or subset of cell types, it may be possible to narrow the visualization to these more narrowly-defined subsets, as done in Section 2.3.3. The problem of identifying scalable alternatives to scatterplots has been extensively studied in the visualization literature. Unfortunately, since ellipse encodings are critical for communicating distortion in the `distortions` package, these scalable alternatives are not immediately applicable. Nonetheless, we expect that future research will identify improved data approximations or graphical encodings. For example, the original data can be replaced with randomized sketches [3, 7], and scatterplots can be replaced with density-based alternatives [1, 5, 2].

2 Comparing embedding and neighborhood stability

Section 2.4 shows that RMetric varies smoothly across hyperparameters. We next study stability with respect to changes in the embedding. We applied t-SNE to the variable density, $\tau = 0$, Swiss roll data (Section 10) using two random initializations and perplexity 10 to induce instability, aligning the results using a Procrustes rotation. Supplementary Fig S1C shows the embeddings with RMetricestimates overlaid.

For sample i, let $\mathbf{H}_1^{(i)}, \mathbf{H}_2^{(i)}$ denote the RMetric estimates across the random seeds, and consider the eigenvalues of $\left(\mathbf{H}_1^{(i)}\right)^{-1}\mathbf{H}_2^{(i)}$, which we write as $\lambda_1^{(i)}, \lambda_2^{(i)}$. We use $\max(|\sqrt{\lambda_1^{(i)}} - 1|, |\sqrt{\lambda_2^{(i)}} - 1|)$ to measure the variability of $\mathbf{H}^{(i)}$ across seeds. This metric captures changes in ellipse size and eccentricity but is rotation invariant. Hence, it lower bounds the instability of RMetricat sample i. To quantify embedding stability, we formed ratios $r_{ij} = \frac{d_2^{(ij)}}{d_1^{(ij)}}$ and $r'_{ij} = \frac{d_1^{(ij)}}{d_2^{(ij)}}$ for all pairs of $K = 15$ nearest neighbors i, j in the original space. For each i, we compute the variances of r_{ij} and r'_{ij} across neighbors j, taking the maximum as our stability measure. Supplementary Fig S1A relates these metrics. Most points lie above the identity, suggesting that RMetric is less stable than the embeddings themselves. The positive association indicates that RMetric instability tracks embedding instability. Supplementary Fig S1B highlights an association between RMetric and position along the roll, with more stability at lower ranges of t.

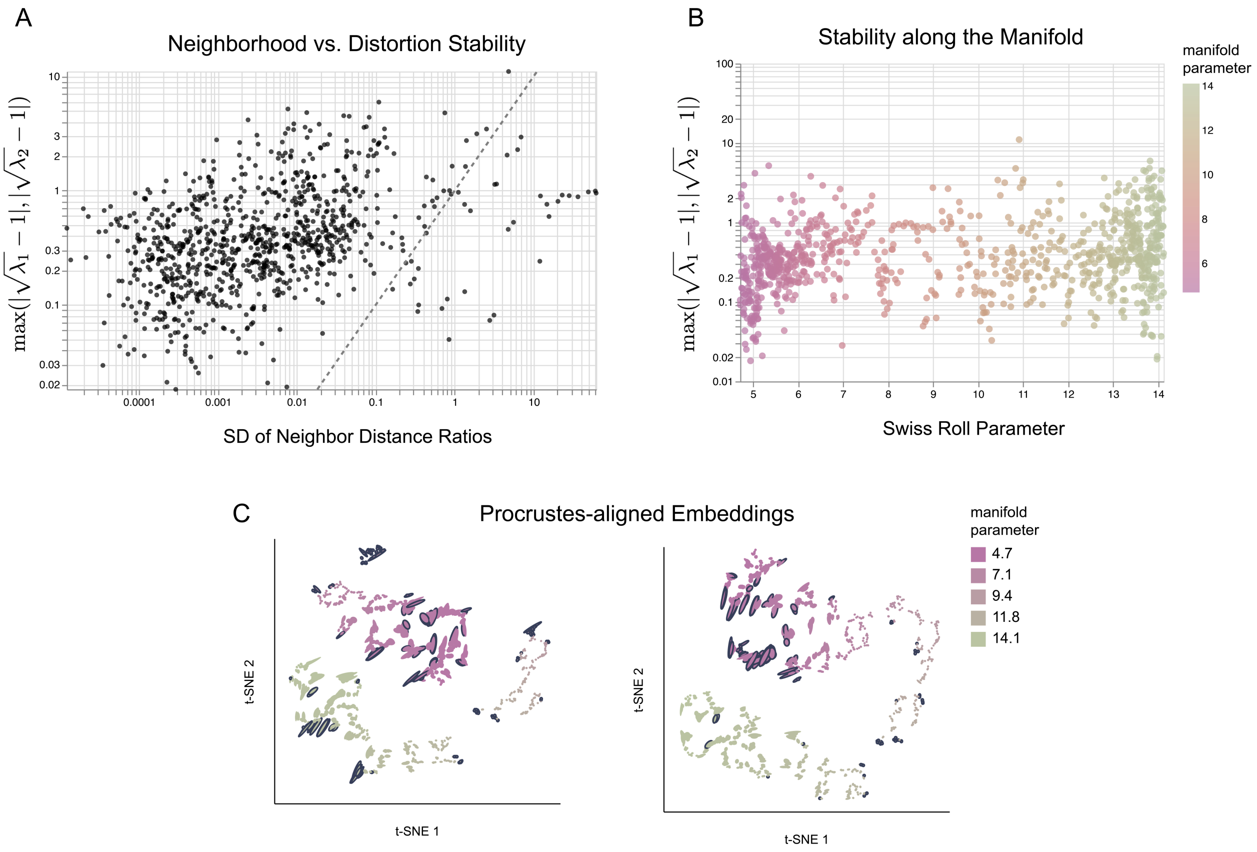

Figure S1: Stability across t-SNE initializations. A. The x-axis is embedding stability, measured by the variance in ratios of neighbor-neighbor distances across embeddings derived from two initializations. The y-axis quantifies RMetric instability using singular values of $\left(\mathbf{H}_1^{(i)}\right)^{-1}\mathbf{H}_2^{(i)}$. Embedding and RMetric stability are positively associated. B. RMetric instability across the Swiss roll parameter t. C. The t-SNE embeddings after Procrustes alignment.

3 Additional baseline comparisons

We compared `distortions` to neMDBD [4] and Sleepwalk [6] algorithms applied to the Gaussian mixture and PBMC data from Sections 2.1.1 and 2.1.2. To accelerate neMDBD on the PBMC data, we subsampled down to 1000 cells. We ran Sleepwalk and `distortions` on the full datasets. The corresponding runtimes are presented in Table S1.

In the Gaussian mixture example, mousing over the two mixture components reveals the density preservation failure. In the embedding space, the clusters appear similar in width, but hovering over the right-hand cluster highlights its smaller interpoint distances (Fig S3A-B). This is the small, high-density cluster from the original data. When using neMDBD, this high-density cluster is associated with larger perturbation scores (Fig S2). The high-density cluster has spread farther in the embedding space.

In the PBMC example, Sleepwalk's color encoding occasionally spaces color so widely that all but the nearest neighbor point appear identical (Fig S3C). Hovering over other points appropriately highlights neighboring cell types (Fig S3D). The neMDBD algorithm identifies monocytes with elevated perturbation scores (Fig S2), matching the larger ellipses in Fig 4A from the `distortions` package. Boundary points between some cell types (bright pink) also draw attention to perturbation sensitivity.

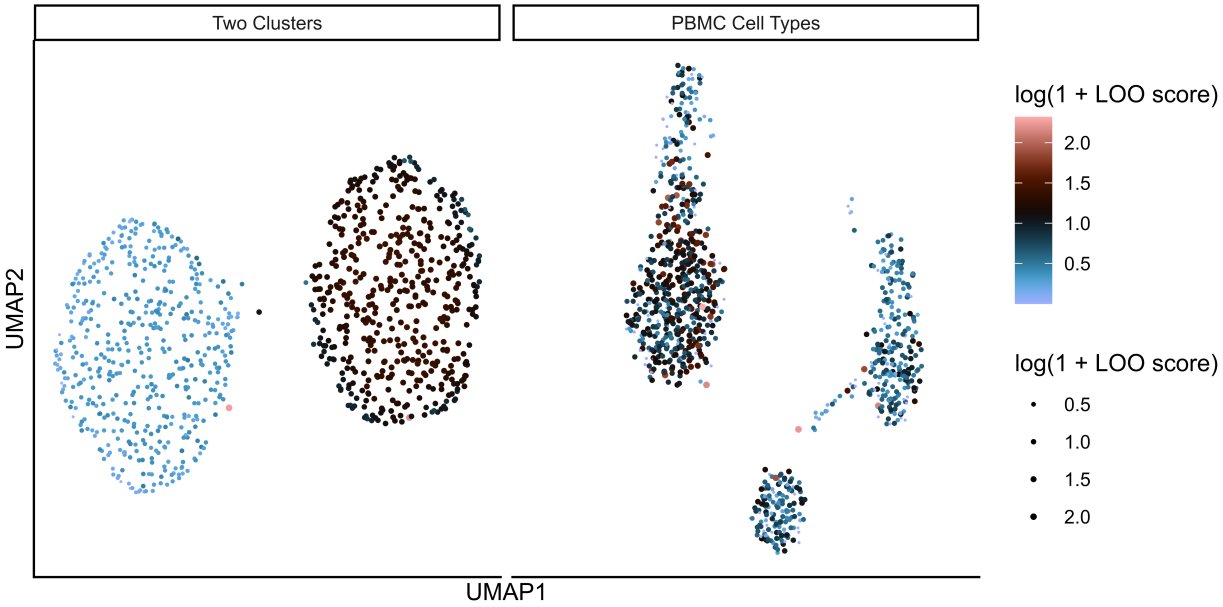

Figure S2: Perturbation scores from the neMDBD algorithm applied to the gaussian mixture simulation and the PBMC data from Sections 2.1.1 and 2.1.2, respectively. In the simulation example, the high density cluster has systematically higher perturbation scores. In the PBMC atlas, a subset of monocytes appears to have higher perturbation on average, and outlying perturbation scores appear near the cell type boundaries.

4 Supplementary tables and figures

References

[1] S. Bachthaler and D. Weiskopf. Continuous scatterplots. *IEEE Transactions on Visualization and Computer Graphics*, 14(6):1428–1435, November 2008.

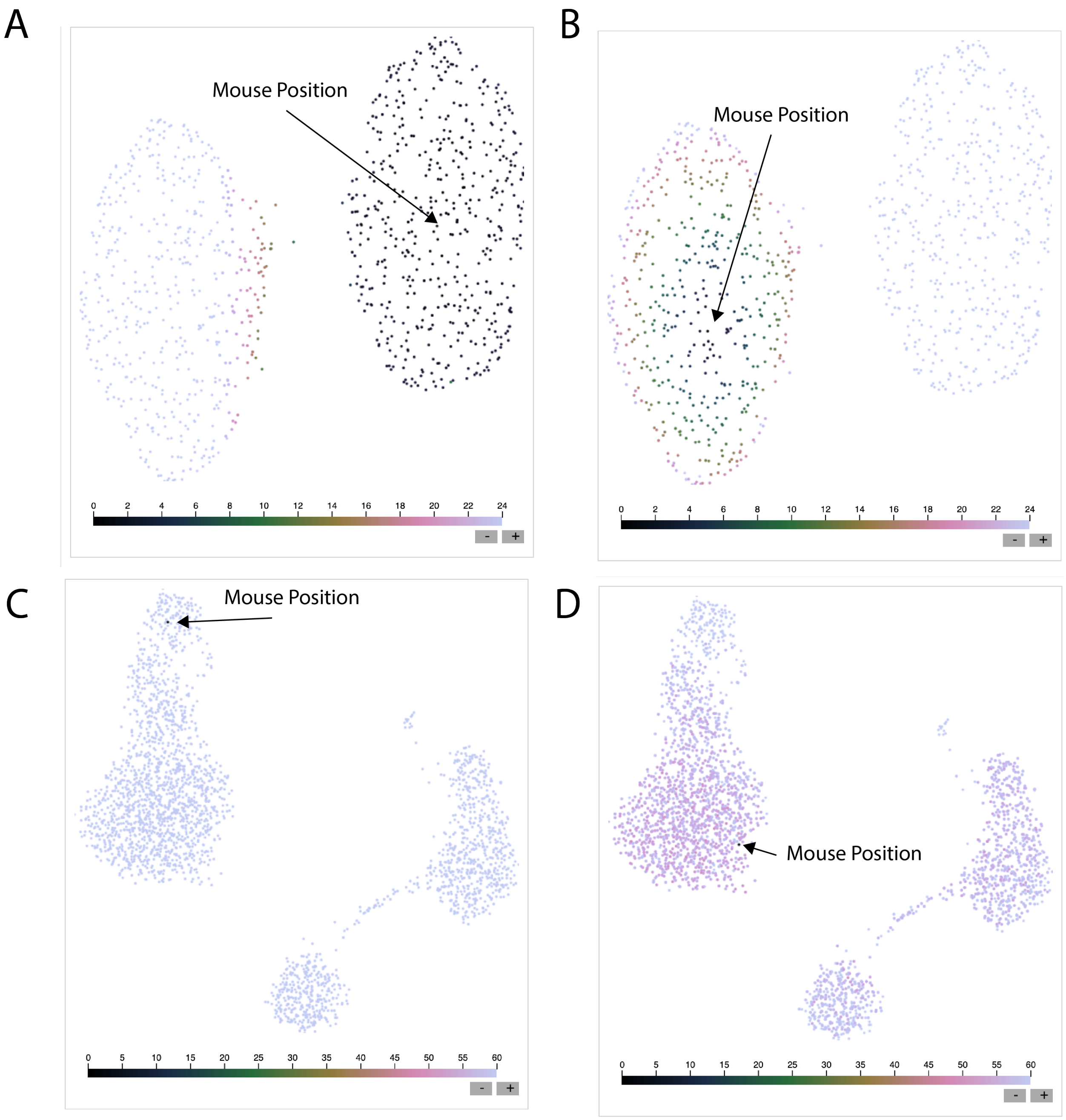

Figure S3: Sleepwalk applied to the Gaussian mixture and PBMC atlas examples. Between panels A and B, the mouse position switches from the high to the low density cluster. Between panels C and D, the mouse position switches from the dendritic to a subset of monocyte cell types.

Method	Dataset	Seconds
distortions	Gaussian Mixture	0.181
neMDBD	Gaussian Mixture	1234.304
sleepwalk	Gaussian Mixture	0.016
distortions	PBMC	0.464
neMDBD	PBMC	1030.709
sleepwalk	PBMC	0.030

Table S1: Runtime for methods considered in Supplementary Section 3. As in Table S2, the neMDBD package was run with parameter `approx=2`.

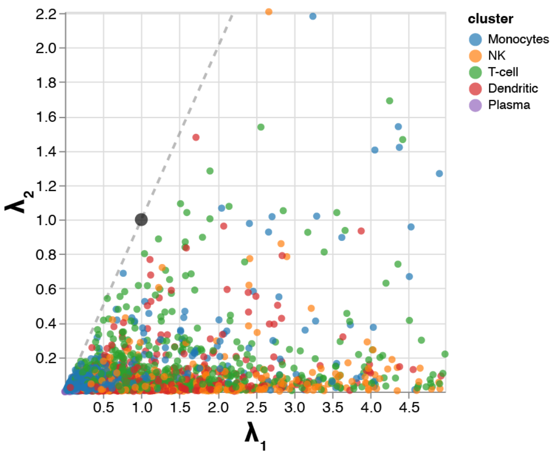

Figure S4: A zoomed-in version of Fig 4D. We have restricted to cells with $\lambda_j^{(i)} < 1$. A second mode of smaller, less eccentric monocytes is visible in this view and contrasts with those that occupy the top right region of Fig 4D. We also see a small cluster of dendritic cells with singular values near the origin, corresponding to the small cluster placed near T cells in Figure 4A-C.

[2] Xin Chen, Yunhai Wang, Huaiwei Bao, Kecheng Lu, Jaemin Jo, Chi-Wing Fu, and Jean-Daniel Fekete. Visualization-driven illumination for density plots. *IEEE Transactions on Visualization and Computer Graphics*, 31(2):1631–1644, February 2025.

[3] Brian Hie, Hyunghoon Cho, Benjamin DeMeo, Bryan Bryson, and Bonnie Berger. Geometric sketching compactly summarizes the single-cell transcriptomic landscape. *Cell Systems*, 8(6):483–493.e7, June 2019.

[4] Zhexuan Liu, Rong Ma, and Yiqiao Zhong. Assessing and improving reliability of neighbor embedding methods: a map-continuity perspective. *Nature Communications*, 16(1), May 2025.

[5] Adrian Mayorga and Michael Gleicher. Splatterplots: Overcoming overdraw in scatter plots. *IEEE Transactions on Visualization and Computer Graphics*, 19(9):1526–1538, September 2013.

[6] Svetlana Ovchinnikova and Simon Anders. Exploring dimension-reduced embeddings with sleepwalk. *Genome Research*, 30(5):749–756, May 2020.

[7] Laura H Tung and Carl Kingsford. Practical selection of representative sets of rna-seq samples using a hierarchical approach. *Bioinformatics*, 37(1):i334–i341, July 2021.

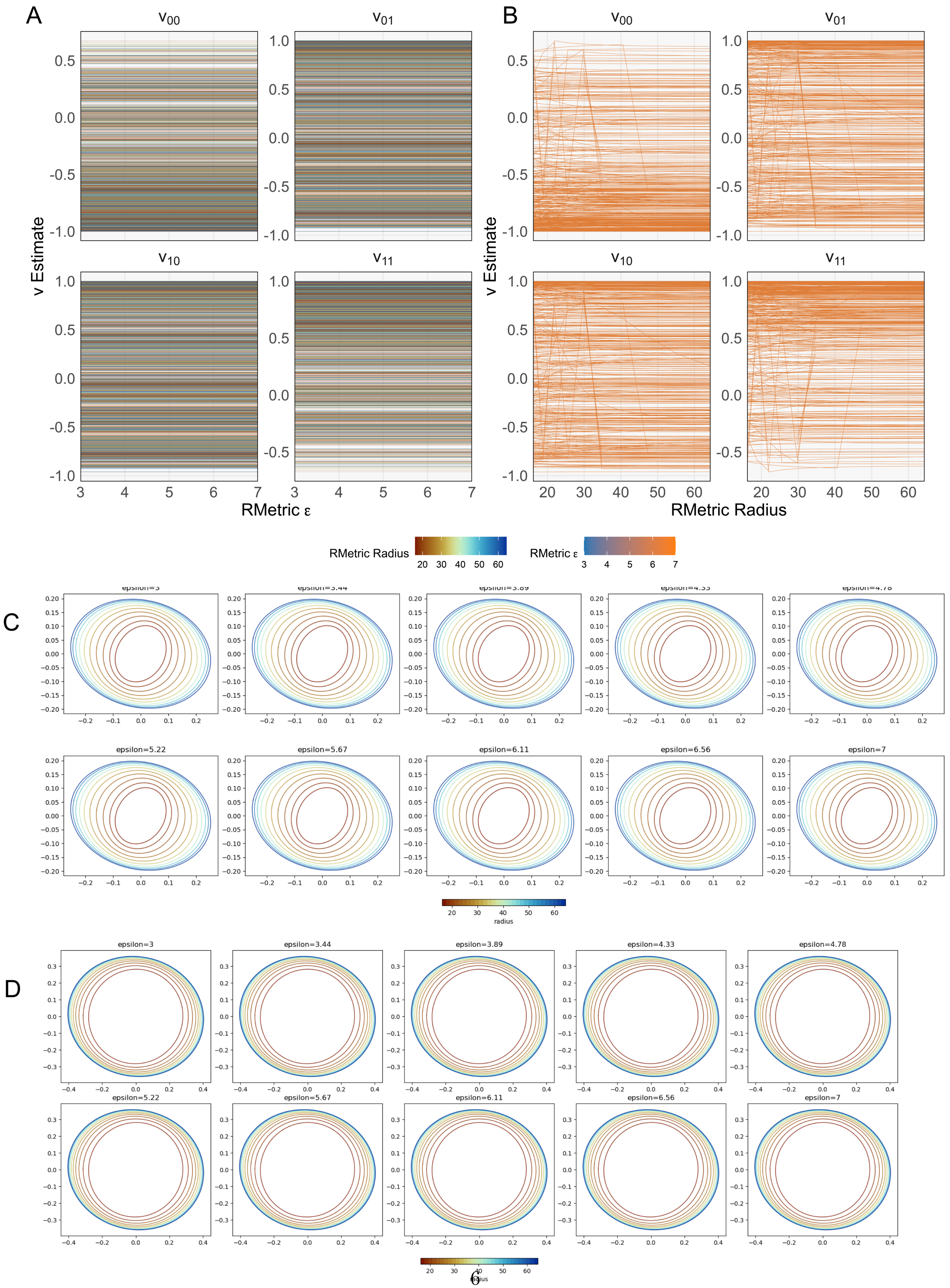

Figure S5: Sensitivity of singular vectors of $\mathbf{H}^{(i)}$ across hyperparameter choices. These are derived from the same experiment as Fig 9. Each line in panels A - B corresponds to one embedding point. While singular vectors appear insensitive to changes in ϵ, they sometimes appear to jump across small changes in radius. Panels C - D investigate these points more closely. These panels show the two samples with the largest absolute difference in singular vector coordinate values across neighboring values of r. Each subpanel within

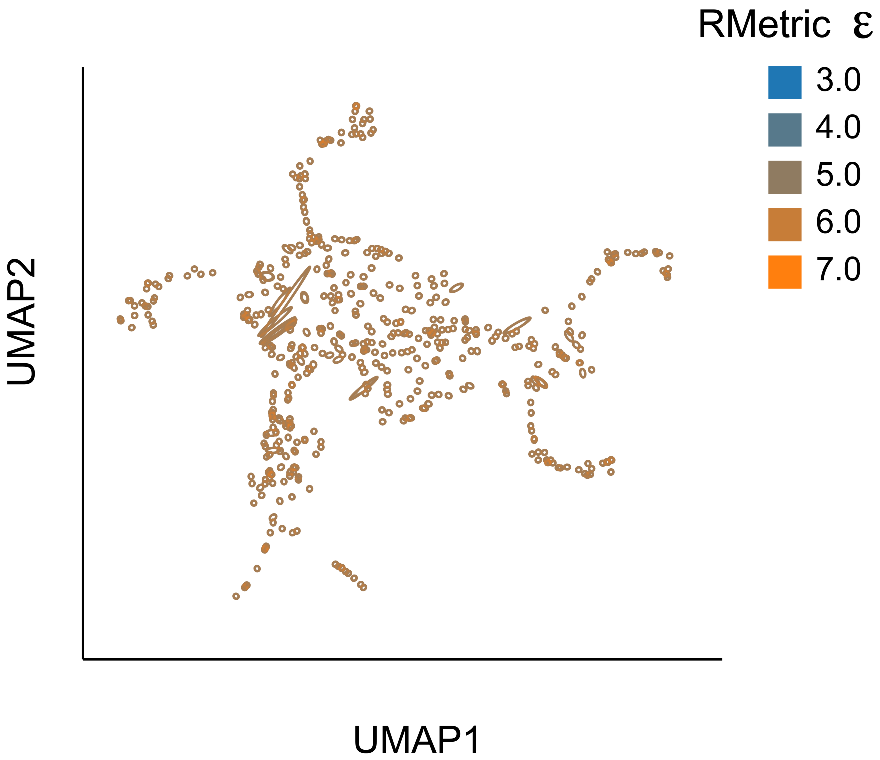

Figure S6: The analog of Fig 9E where ellipse borders encode the RMetric radius hyperparameter. Radius has a weaker effect on ellipse size and eccentricity than ϵ.

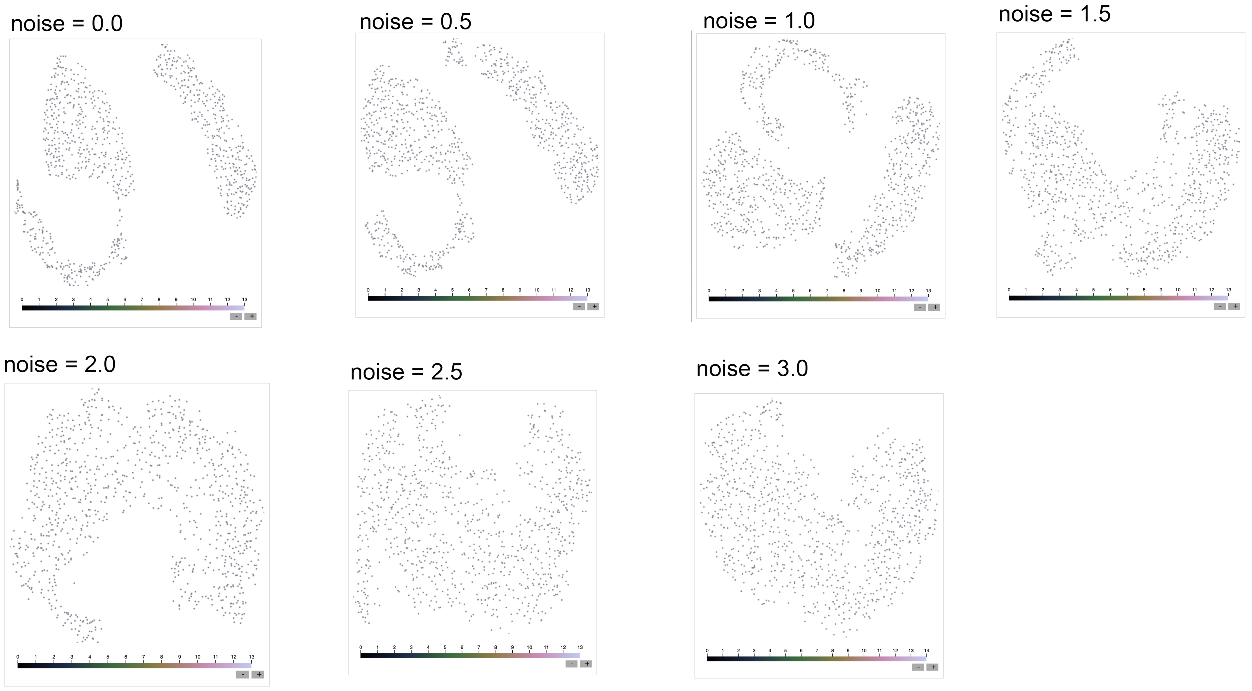

Figure S7: Sleepwalk visualization of the noisy, variable density Swiss roll across noise levels τ. Updated views after interaction are shown in Supplemental Fig S8.

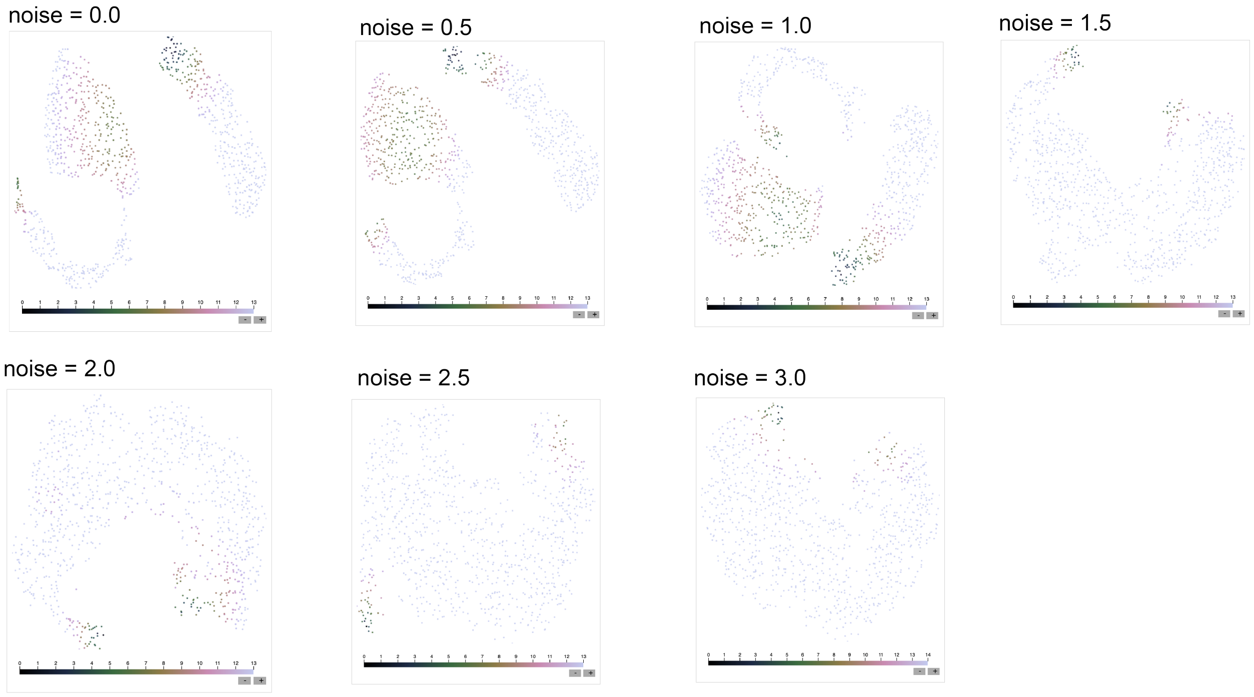

Figure S8: A version of Supplemental Fig S7 after placing the Sleepwalk cursor over a fragmented region of the t-SNE embedding. Darker blue points are close the nearest hovered point in the original data space. The size of the embedding region with low distance to hovered point in the original data space reflects a failure to preserve density.

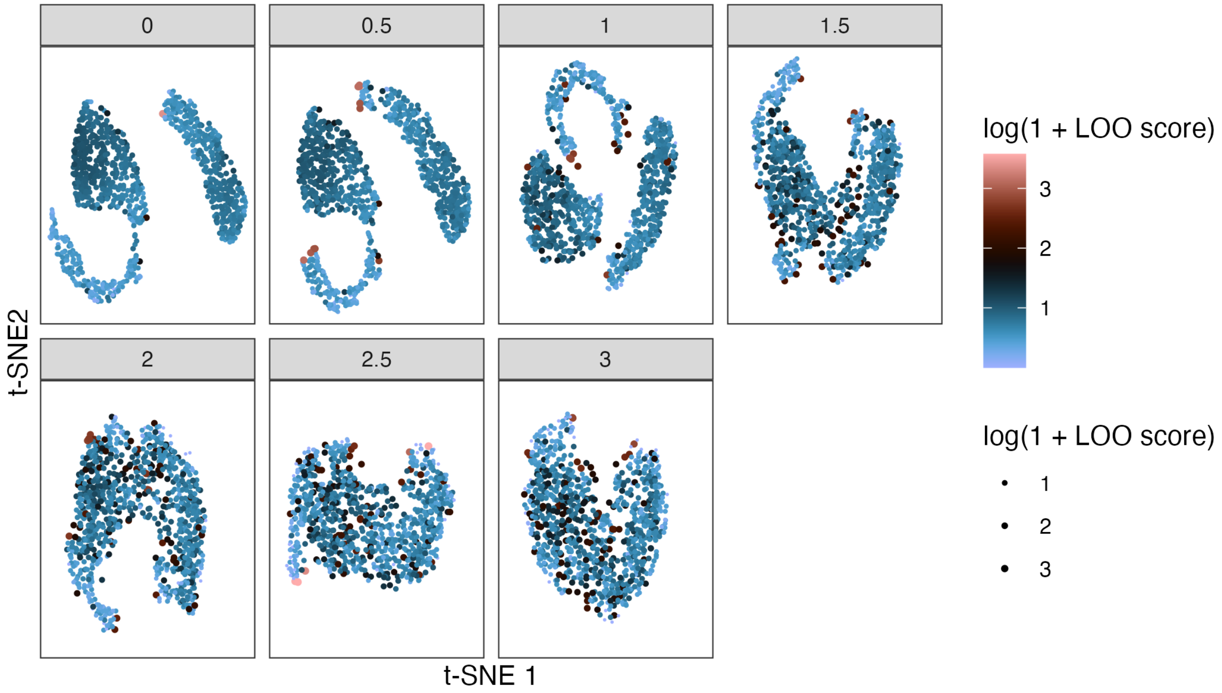

Figure S9: LOO perturbation scores applied to the noisy, variable density Swiss roll noise levels. Scores are shown on a $\log(1+x)$ scale so that extreme outlier scores do not drown out other variation in LOO perturbation score. Perturbation scores slightly increase among points in the original data space's high-density regions. As the noise level τ increases, a larger fraction of points have above average perturbation scores, but the maximum scores are less extreme.

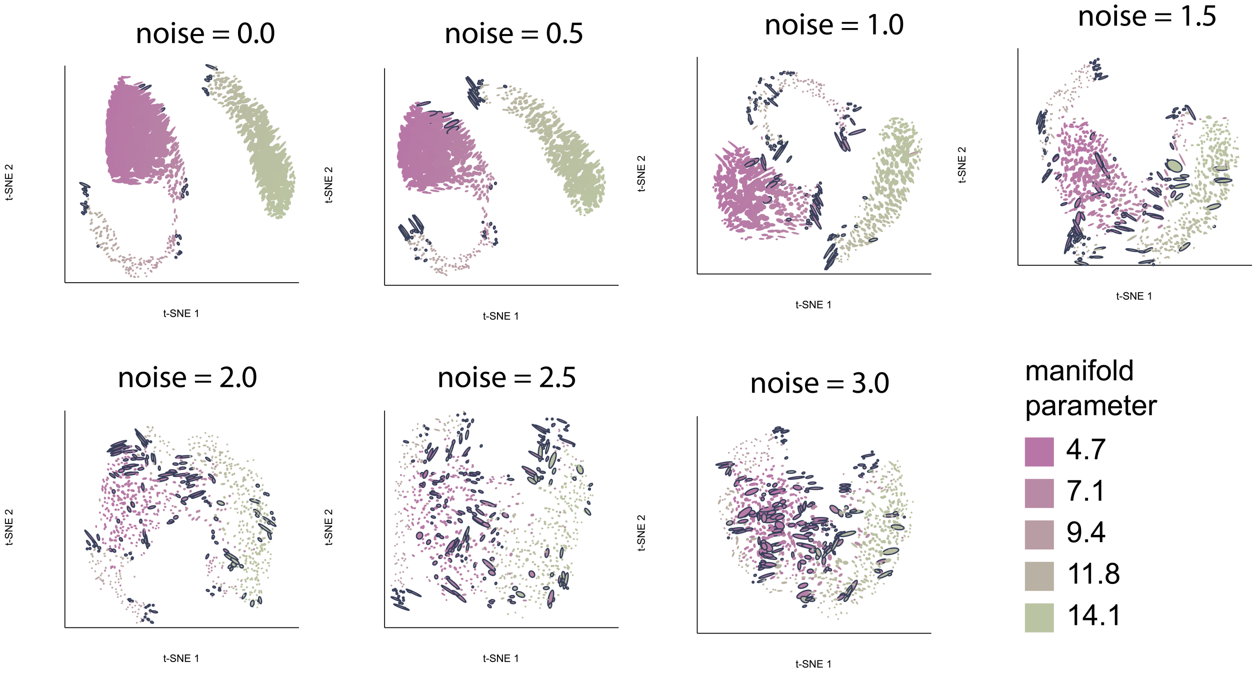

Figure S10: Initial views of the `distortions` fragmented neighborhoods visualization for *t*-SNE embeddings of the noisy, variable density Swiss roll, before placing the cursor over the panels. Difference in ellipse size reflect the failure for *t*-SNE to preserve the original data density in the embedding.

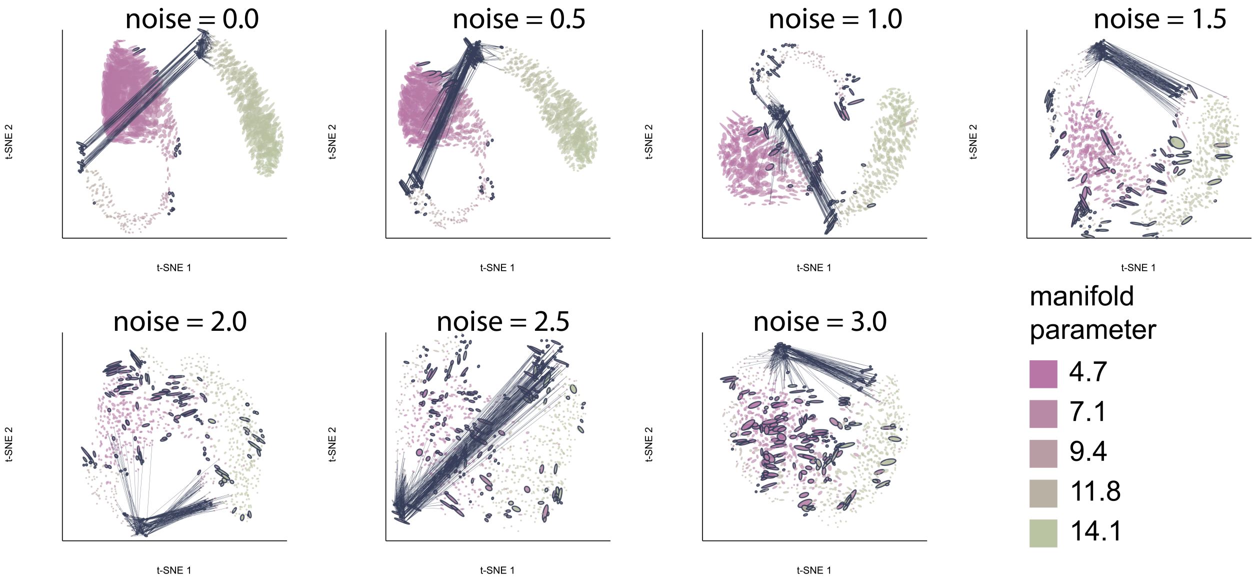

Figure S11: A version of Supplemental Fig S10 after hovering over example fragmented neighborhoods highlighted in the initial view. In addition to the density preservation failure evident in that view, we can identify discontinuities in the embedding map.

**Method**	**Mean (seconds)**	**SD (seconds)**
distortions	0.93	0.05
neMDBD	1156.96	27.55
sleepwalk	0.04	0.02

Table S2: Runtime for methods considered in Section 2.5, averaged across noise levels τ. The neMDBD package was run with parameter `approx=2` to accelerate leave-one-out computation.
